# Development of a gene panel for immune status assessment in sepsis

**DOI:** 10.1186/s13613-025-01594-1

**Published:** 2025-10-27

**Authors:** Chao Gao, Xinxing Lu, Yiwei Jiang, Ying Tang, Yunhui Ni, Hanbing Chen, Xiaojing Wu, Xing Zhou, Yi Yang, Ling Liu, Jie Chao, Jianfeng Xie, Haibo Qiu

**Affiliations:** 1 Jiangsu Provincial Key Laboratory of Critical Care Medicine, Department of Critical Care Medicine, Zhongda Hospital, School of Medicine, Southeast University, Nanjing, 210000 China; 2 School of Life Science and Technology, Southeast University, Nanjing, 210000 China; 3 Department of Physiology, School of Medicine, Southeast University, Nanjing, 210000 China

**Keywords:** Sepsis heterogeneity, Stratification, Endotype, Immunotherapy, Transcriptomics

## Abstract

**Background:**

Sepsis is characterized by a dysregulated immune response to infection, with a balance between hyperinflammation and immunosuppression, which determines the patient's immune status. Real-time monitoring of the immune status in sepsis is crucial for guiding immunotherapy. However, reliable biomarkers are lacking. This study aims to identify a panel of biomarkers for rapid bedside assessment of immune status in sepsis to guide immunotherapy decisions.

**Results:**

*TBX21, GNLY, PRF1*, and *IL2RB* represent the immune status in sepsis. These genes demonstrated discriminatory power in the external validation, with area under the curve values ranging from 0.891 to 0.909 across several machine learning models. 99 double-blind randomized patients with sepsis were clustered into two endotypes on the basis of the expression of the four-gene panel. Higher 90-day mortality was observed in patients with sepsis treated with hydrocortisone (Odds ratio 12.46, 95% confidence intervals 3.11 to 65.72) or thymosin (Odds ratio 4.17, 95% confidence intervals 1.13 to 16.51) within the high-expression 4-gene panel endotype, but not in another endotype.

**Conclusions:**

The results support the potential utility of a four-gene panel to assess immune status and guide immunotherapy; further prospective validation and translational studies are warranted.

*Trial registration* National Medical Research Registration and Filing Information of China, 2022ZDSYLL196-P01. Registered 26 May 2023, https://www.medicalresearch.org.cn/login

**Supplementary Information:**

The online version contains supplementary material available at 10.1186/s13613-025-01594-1.

## Introduction

Sepsis is characterized by an unbalanced immune response in which excessive inflammation concurrently occurs with immunosuppression [1]. The interplay between hyperinflammation and immunosuppression defines an individual's immunological endotype. The high degree of individualization of immunological endotypes poses a significant obstacle to diagnosis and personalized treatment. Despite the availability of various immunomodulatory drugs, [2] the high variability in immunological responses in sepsis presents substantial challenges for diagnosis and personalized treatment [3–5]. Therefore, accurate identification of immune status is a critical step toward providing appropriate immunotherapies for sepsis [6]. Some indicators currently used for immune recognition, such as monocyte human leukocyte antigen-DR isoform (HLA-DR), are associated with sepsis-related immunosuppression and are widely used in clinical immunomonitoring [7–9]. However, relying on a single biomarker for sepsis is insufficient to guide immunotherapy that targets hyperinflammation or immunosuppression because each biomarker reflects only a limited number of pathological processes. Sepsis involves the simultaneous activation or suppression of multiple pathways; therefore, a biomarker panel may more accurately capture the immune status.

Stratification of the heterogeneous sepsis population is necessary to determine immune status. Although several studies have stratified sepsis subgroups, most have focused on prognosis (prognostic enrichment strategies) and severity [10–12] rather than directly assessing immune status. Davenport et al. [13] classified sepsis patients into two distinct groups on the basis of sepsis response signatures (SRS1 and SRS2) according to unsupervised hierarchical clustering. The results revealed that SRS1 exhibited immunosuppressive features (T-cell exhaustion and downregulation of HLA class II), and no differences in the expression of the cytokine genes encoding TNF, IL6, or IL1B were noted between the two groups. Another study identified four molecular endotypes (Mars 1–4) associated with sepsis [10]. Additionally, on the basis of multiple transcriptomic datasets, sepsis can be classified into three subtypes: inflammatory (innate immune activation), adaptive (adaptive immune activation), and coagulopathic (coagulation dysfunction) [14]. In addition, sepsis stratification has potential therapeutic implications, with varying effects of α, β, γ, and δ stratification across clinical trials [15]. These studies emphasize the heterogeneous nature of sepsis and how clustering analyses enhance our understanding of the diverse pathophysiological mechanisms of this syndrome.

Stratification of sepsis patients on the basis of immune status has been reported via the use of a 15-gene panel to classify patients into immune-adaptive prevalent (IA-P) and immune-innate prevalent (IN-P) groups. Compared with IN-P patients, IA-P patients presented lower white blood cell counts and shock rates. Additionally, IA-P patients had lower SOFA scores, APACHE II scores, and mortality [16].

We hypothesized that specific gene expression levels correlate with immune status and can serve as biomarkers for stratifying and managing patients with sepsis on an individualized basis. This study aimed to screen a more precise and clinically applicable set of immune status biomarkers associated with sepsis to support immunotherapeutic decision-making.

## Methods and materials

### Biomarker screening and validation for immune status in sepsis

The discovery and validation datasets used in this study were obtained from the Gene Expression Omnibus (https://www.ncbi.nlm.nih.gov/geo/): GSE65682 and GSE154918. The single-sample gene set enrichment analysis (ssGSEA) algorithm was used to measure the enrichment levels of 22 immune signatures in the sepsis samples. The samples were divided into two subtypes on the basis of the median ssGSEA score. The relative proportions of the different immune cell types were estimated via the CIBERSORT deconvolution algorithm [17].

Weighted gene coexpression network analysis (WGCNA) was conducted to select the module with the strongest correlation to the subtypes. The PPI network was used to screen for hub genes in the candidate modules. Machine learning algorithms, including the generalized linear model (GLM), support vector machine (SVM), XGBoost (XGB), and random forest (RF), were used to validate the immune status prediction models. The performance of these models was evaluated via the area under the receiver operating characteristic (ROC) curve (AUC). All algorithms and Kaplan–Meier survival curves were generated via R4.3.1.

### WGCNA analysis

Co-expression networks were constructed in R 4.3.1 with WGCNA. After standard preprocessing, the top 10,000 genes by MAD were retained. We used a signed network and signed TOM with Pearson correlation (corType = "pearson"). Soft-thresholding power was selected by pickSoftThreshold over powers 1–30, prioritizing a high scale-free fit and reasonable connectivity; we chose β = 20 (scale-free R^2^≈0.92; Fig. S2A). Module detection used blockwiseModules with minModuleSize = 30, deepSplit = 2, mergeCutHeight = 0.2, reassignThreshold = 0, numericLabels = TRUE. Module–trait associations were computed by Pearson correlation against the immunity_H/L phenotype (P < 0.05). The dark-red module showing the strongest association with immune status was taken forward for GO/KEGG analysis (Fig. S2D, S2E) and candidate selection.

### PPI network and hub selection

We built a PPI network in STRING (human), restricting evidence channels to Experiments + Databases and retaining high-confidence edges (combined score ≥ 0.70). On the WGCNA candidate set from the immune-associated module (prioritized by |MM| and |GS|, with a permissive inclusion threshold |MM|> 0.80 & |GS|> 0.20), isolated nodes were removed. We computed degree and betweenness centralities and derived a rank-product meta-rank; Top-k (k = 6) nodes were designated PPI hubs. The final panel was defined by the intersection of stringent WGCNA hubs (|MM|> 0.85 & |GS|> 0.50) and PPI hubs. Robustness checks across k = 5–10 and score = 0.6–0.9 yielded stable selections.

### External validation cohorts and patients

For external validation, we used two independent cohorts:

- (1) Public transcriptomic dataset (GSE154918): we restricted analyses to 39 adult patients diagnosed with sepsis or septic shock, which were used for case–case comparisons and external validation of model discrimination.
- (2) Prospective clinical cohort (Zhongda Hospital): 99 randomized, double-blind sepsis patients were enrolled and profiled for the four-gene panel to derive immune-status endotypes (ISS1/ISS2) and to explore associations with treatment response. Demographic and clinical characteristics are summarized in Table 1.

**Table 1:** Baseline clinical and demographic characteristics of the external validation cohort, all and stratified by ISS gene-panel endotypes (ISS1 vs ISS2)

	All (n = 99)	ISS1 (n = 52)	ISS2 (n = 47)	P value
Demographics				
Gender (females)^*^	40 (40.40)	19 (36.54)	21 (44.68)	0.4097^a^
Age (years)^#^	72 (61, 81)	68 (59, 79)	76 (67, 84)	0.0358^b^
BMI^#^	23.4 (21.1, 25.7)	23.7 (21.0, 26.0)	23.4 (21.4, 25.3)	0.7601^b^
Vital Signs				
Body temperature (°C)^#^	37.5 (37.0, 38.1)	37.3 (37.1, 38.4)	37.5 (37.0, 38.0)	0.9590^b^
Severity Scores				
SOFA score^#^	10 (8, 12)	9 (6, 11)	11 (9, 12)	0.0517^b^
APACHE II^#^	20 (15, 25)	20 (15, 25)	20 (17, 27)	0.6480^b^
Clinical Laboratory Results				
Blood pH^#^	7.37 (7.30, 7.41)	7.35 (7.29, 7.40)	7.38 (7.31, 7.41)	0.4325^b^
PaO2/FiO2^#^	233.8(168.6, 311.7)	222.9 (175.6, 331.4)	253.8 (168.3, 305.4)	0.3520^b^
Lymphocyte (G/L)^#^	0.58 (0.36, 0.86)	0.57 (0.36, 0.90)	0.59 (0.37, 0.80)	0.9704^b^
Neutrophil (G/L)^#^	13.10 (6.79, 17.30)	13.75 (6.94, 18.10)	11.81 (6.52, 16.25)	0.2932^b^
Monocyte (G/L)^#^	0.48 (0.17, 0.81)	0.49 (0.22, 0.90)	0.48 (0.16, 0.65)	0.2882^b^
Treatment or Respiratory support				
MV^*^	80 (80.81)	42 (80.77)	38 (80.85)	0.9918^a^
Hydrocortisone^*^	40 (40.40)	19 (36.54)	21 (44.68)	0.4097^a^
Thymosin^*^	23 (23.23)	9 (17.31)	14 (29.79)	0.1725^a^
Complications / Prognosis				
Septic shock^*^	74 (74.75)	36 (69.23)	38 (80.85)	0.1839^a^
Pulmonary infection^*^	22 (22.22)	10 (19.23)	12 (25.53)	0.4514^a^
LOS in ICU (days)^#^	6 (3, 11)	7 (3, 14)	4 (3, 9)	0.1258^b^
28-day mortality^*^	28 (28.28)	14 (26.92)	14 (29.79)	0.7520^a^
90-day mortality^*^	32 (32.32)	16 (30.77)	16 (34.04)	0.7280^a^

^*^Frequency (%)

^#^Median (IQR)

*a* Chi-square test

*b* Mann–Whitney U test

*MV* mechanical ventilation, *LOS* length of stay, *SOFA* Sequential organ failure assessment

All human samples used in this study were obtained from the Department of Critical Care Medicine, Zhongda Hospital, Southeast University, and were approved by the Ethics Committee of Zhongda Hospital (Approval No. 2022ZDSYLL196-P01). Written informed consent was obtained from all the participants. The patients included in this study met the diagnostic criteria for Sepsis 3.0, and treatment allocation was randomized and double-blinded. The following exclusion criteria were applied: (1) < 18 years old; (2) sepsis diagnosed > 24 h prior; (3) transferred from another intensive care unit (ICU); (4) a second admission to an ICU within 30 days; (5) hematological malignancy or autoimmune disease; (6) thrombocytopenia or unknown platelet count within 24 h of ICU admission; and (7) cirrhosis, hepatitis, or history of splenectomy.

### Cecal ligation puncture model

All animal procedures adhered strictly to the National Institutes of Health Guidelines for the Use of Laboratory Animals. The research protocol was approved by the Institutional Animal Care and Use Committee at the medical school of Southeast University, Jiangsu, China (Permit Number: 20190222014). The mouse cecal ligation puncture (CLP) procedure was performed according to a previously reported protocol, and sepsis models of varying severity were established by selecting 5%/20% of the ligation length [18].

### Real-time PCR

Total RNA was extracted via TRIzol reagent (TaKaRa, Biotechnology, China). cDNA synthesis was performed via HiScript II Q RT SuperMix for qPCR (Vazyme, Vazymebiotech, China). Real-time PCR was performed using AceQ Universal SYBR qPCR Master Mix (Vazyme).

### Model evaluation and ROC analysis

We defined Immunity_H as the positive class. For each model, predicted probabilities were obtained on the held-out internal test set and on the independent external cohort. ROC curves were generated from these probabilities (higher values indicate Immunity_H), and AUCs with DeLong 95% confidence intervals were computed (pROC). Cross-validation (repeated fivefold, 10 repeats) was used only for hyperparameter tuning; final ROC/AUC were computed on held-out data. A single probability cutoff was pre-specified on the internal test set by maximizing Youden’s J and then fixed for external validation. At this fixed cutoff we report sensitivity, specificity, and accuracy (with exact binomial 95% CIs for sensitivity and specificity) and provide the confusion matrices (TN/FP/FN/TP); results at a 0.5 cutoff are additionally listed as a reference.

### Regression analysis and verification

Primary frequentist analysis (reported in the main text). We fit frequentist logistic regression models in GraphPad Prism (MLE with Wald confidence intervals) for 90-day/28 day mortality within the ISS2 stratum, with treatment exposure (hydrocortisone or thymosin; separate models) as the predictor. These estimates are presented in the main text.

Bayesian robustness verification (reported in the Supplement). To address small-sample uncertainty and potential separation, we additionally fit Bayesian logistic regression (logit link) with weakly informative priors (Student-t(df = 3, 0, 2.5) on the treatment coefficient; Student-t(df = 3, 0, 10) on the intercept). We summarize posterior median odds ratios with 95% credible intervals (CrIs), the posterior probability Pr(OR > 1), and g-computed posterior standardized risks for exposed (p₁) and unexposed (p₀) along with the risk difference (RD) and its 95% CrI. Posterior predictive checks and leave-one-out cross-validation (LOO-CV) were used to assess model adequacy. All Bayesian outputs are provided in the Supplement (Table S7: hydrocortisone; Table S8: thymosin). Intervals from Bayesian models are denoted CrIs to distinguish them from frequentist CIs.

## Methods–statistical analysis

### Outcomes

The primary outcome was 90-day mortality. The secondary outcome was 28-day mortality.

### Statistical analysis

All analyses were performed with GraphPad Prism 9.5 and R 4.3.1. Continuous variables were summarized as median [IQR] because most measures were right-skewed, and between-group comparisons were therefore conducted primarily with the Mann–Whitney U test (two-sided). Categorical variables were summarized as n (%) and compared using the chi-square test when all expected cell counts ≥ 5; otherwise, two-sided Fisher’s exact tests were used for low/sparse 2 × 2 tables. Survival was analyzed by Kaplan–Meier methods and compared with the log-rank test. Correlations between approximately normally distributed variables were assessed with Pearson’s r, and with Spearman’s ρ otherwise. A two-sided α = 0.05 defined statistical significance. Treatment × endotype effect modification in Cox models by including a product term; significance was assessed using two-sided Wald tests and likelihood-ratio tests (models with vs without interaction). Proportional-hazards assumptions were assessed by Schoenfeld residuals.

## Results

### Screening for a gene panel associated with sepsis immune status

**Fig. 1:**
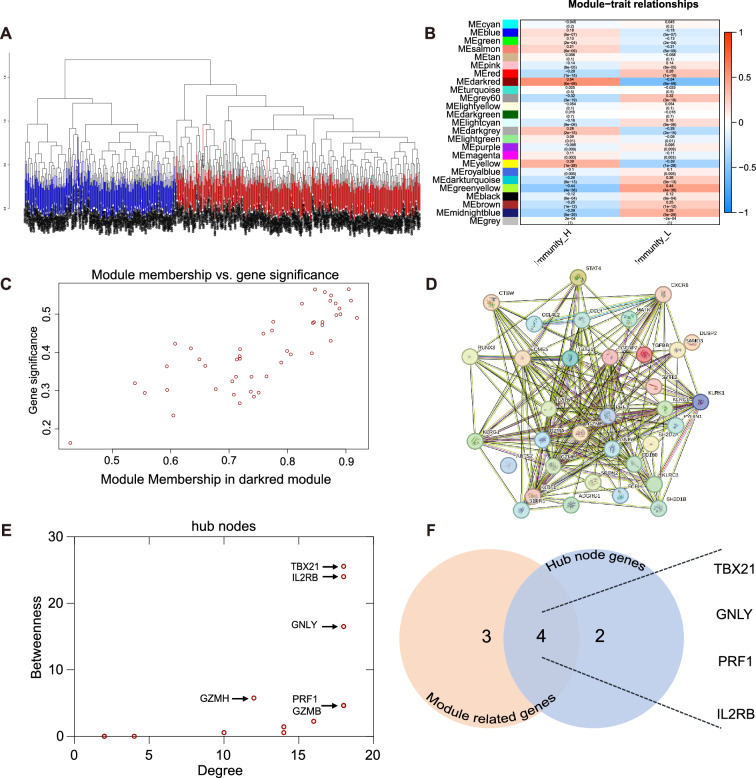
Sample stratification based on ssGSEA and validation. **A** Samples belonging to the high immune score subgroup are shown in red (n = 469, 61.71%), and low score samples are shown in blue (n = 291, 38.29%). **B** The correlation between the module eigengene and sepsis immune status. **C** Seven genes within high thresholds (MM > 0.85, GS > 0.5) were selected. **D**, **E** Low thresholds (MM > 0.80, GS > 0.2) were used to select genes to construct a PPI network, and six hub nodes were selected on the basis of degree and betweenness. **F** The intersection of the two groups included four genes. *GS*  gene significance, *MM* module membership, *PPI* protein–protein interaction, *ssGSEA* single-sample gene set enrichment analysis

ssGSEA was performed to calculate the immune infiltration scores of 760 sepsis samples. On the basis of the median ssGSEA score, the samples were classified into two subtypes: high (immunity_H) and low (immunity_L) (Fig. 1A, S1). To explore the biological characteristics of these immune subtypes, WGCNA was performed to construct co-expression networks and identify gene modules that were strongly correlated across samples. Pearson’s correlation analysis was applied to evaluate the module-trait relationships (Fig. 1B), with the dark red module showing the strongest correlation with immune status (Cor = 0.54, P = 6e-59). The genes in this module were primarily associated with T- and NK-cell immunity (Figure S2D and S2E).

A high threshold (MM > 0.85, GS > 0.50) was subsequently applied to identify seven genes (*EOMES, PRF1, FGFBP2, TBX21, IL2RB, RUNX3*, and *GNLY*) that were strongly correlated with both modules and traits (Fig. 1C). Concurrently, a lower threshold (MM > 0.80, GS > 0.20) was used to select genes to construct a PPI network. Six genes were identified: *TBX21, IL2RB, GNLY, PRF1, GZMB*, and GZMH (Fig. 1D and E). Four overlapping genes from the two groups, which were expressed primarily in NK and T cells (Figure S3), were established as sepsis immune status biomarkers (Fig. 1F). To explore the relationship between the gene panel and traditional immune markers, we analyzed RNA-seq data from patients with sepsis at our hospital. However, *TBX21* was not detected in the sequencing data. Therefore, we focused on three other genes: *PRF1*, *IL2RB*, and *GNLY*. Significant correlations were observed between the expression of these genes (*PRF1*, *IL2RB*, and *GNLY*) and monocyte HLA-DR expression and the percentage of NK cells (Figure S5).

### Validating the predictive efficacy of the gene panel for immune status

**Fig. 2:**
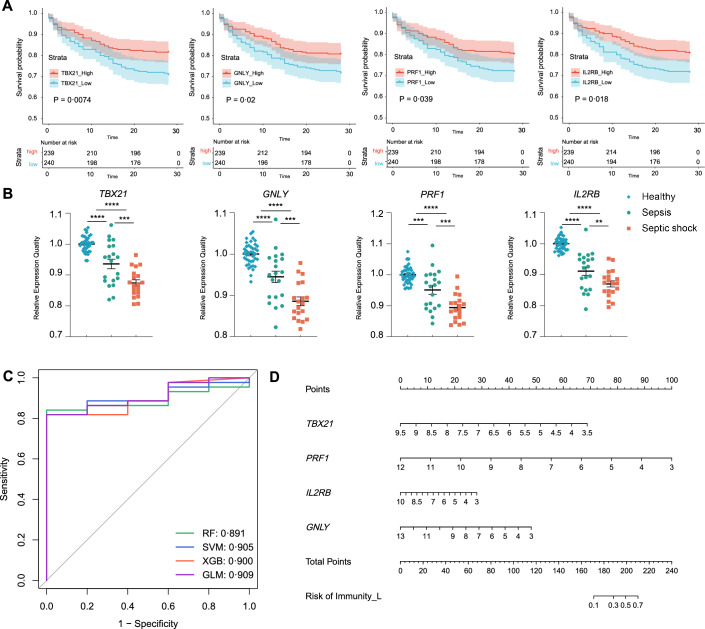
Validation of gene panel for the identification of immune status in sepsis. **A** Kaplan–Meier survival curves for the four ISS genes. **B** Validation of the ISS gene expression via an external dataset (GSE154918). **C** The predictive performance of the different models was evaluated with ROC curves. **D** Construction of a nomogram model based on the ISS-gene panel. *ROC* receiver operating characteristic *P < 0.05, **P < 0.01, ***P < 0.001, and ****P < 0.0001 compared with the control

To validate the prognostic role of the gene panel, we conducted survival analyses of the four genes via clinical data from the GSE65682 dataset (Fig. 2A). Moreover, the external validation dataset GSE154918 was used to verify changes in gene panel expression between sepsis patients and healthy individuals. The gene panel expression levels were significantly different between the above two groups (Fig. 2B). Notably, differences were also observed between sepsis and septic shock, implying that the four genes were not only downregulated in sepsis but also correlated with sepsis severity (Fig. 2B), and the CLP mouse model demonstrated similar differences (Figure S4C).

To evaluate the potential of the gene panel to identify the immune status in sepsis patients, four machine learning algorithms, GLM, SVM, XGB, and RF, were used to construct predictive models. The GSE65682 dataset was split by stratified random sampling (70%/30% training/test; fixed seed), and hyperparameters were optimized on the training set using repeated fivefold cross-validation (5 × 10) with ROC-AUC as the selection metric. The independent external dataset GSE154918 was used only to validate the models. ROC curves were generated to assess the predictive performance of each model (Fig. 2C). For classification metrics on the external cohort, we applied the pre-specified probability cutoffs chosen on the internal test set by maximizing Youden’s J (i.e., fixed cutoffs carried to external validation), and reported sensitivity, specificity, and accuracy (Table S2); exact binomial 95% CIs and confusion matrices are provided in the Supplement (Table S3). Discrimination on the external cohort remained strong (AUC, DeLong 95% CI): RF 0.891 (0.764–0.991), SVM 0.905 (0.812–0.997), XGB 0.900 (0.810–0.990), and GLM 0.909 (0.809–1.000). At the fixed internal cutoffs, specificity was 1.000 across models with sensitivities ranging 0.432–0.818 and accuracies 0.490–0.837 (Table S2).Finally, a nomogram based on the GLM was created (Fig. 2D), with sensitivity and specificity derived from the model’s predicted probabilities via ROC analysis, demonstrating a reliable approach for predicting individual immune status and guiding tailored adjuvant treatment in sepsis patients.

We further attempted to use this gene panel, along with patient age and sex, to predict multiorgan failure (MOF) 3 days after ICU admission (detailed cohort acquisition was described in Methods). Despite an AUC exceeding 0.7 (Figure S7), the predictive efficacy for multiorgan failure was lower than its performance in predicting immune status, consistent with its design as an immune-status marker. Because MOF is a multifactorial downstream outcome influenced by hemodynamic, metabolic, infectious, and iatrogenic factors, only partial carry-over from immune status to short-term MOF risk is expected.

### ISS-Gene panel identifies subgroups with elevated mortality risk from hydrocortisone or thymosin treatment

**Fig. 3:**
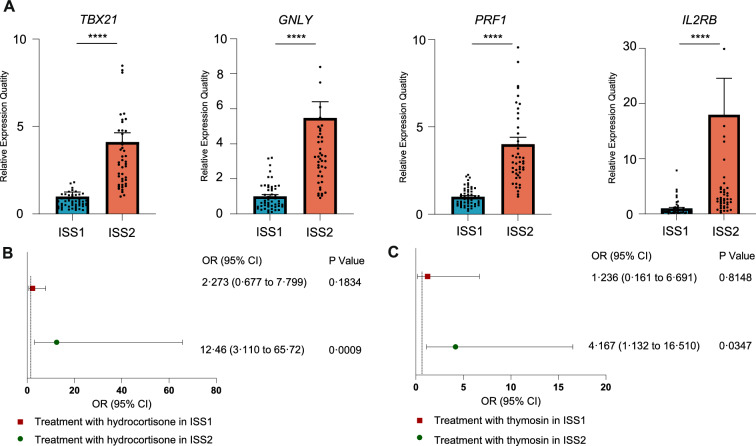
Different responses of ISS1 and ISS2 patients to hydrocortisone or thymosin treatment. **A** Differences in the expression of four gene panels between ISS1 and ISS2. **B** Hydrocortisone or thymosin therapy higher 90-day mortality in the ISS2 subgroup but not in the ISS1 subgroup

To assess the clinical relevance of the gene panel, the mRNA levels of the target genes were examined in 99 peripheral blood samples from double-blind, randomized patients with sepsis. Consensus clustering on the basis of the four-gene panel expression profiles was used to categorize the samples based on two distinct immune statuses in sepsis (ISS, Table 1, Figure S6). ISS1, characterized by low expression of the four immune-related genes (Fig. 3A), represented a sepsis subgroup with an immunosuppressive endotype. In the external cohort (n = 99), median age was higher in ISS2 than ISS1 (76 vs 68 years, P = 0.0358). SOFA scores showed a non-significant trend toward higher values in ISS2 (11 vs 9, P = 0.0517). Apart from these variables, baseline characteristics were broadly similar (Table 1). These findings indicate that ISS stratification differentiates immune endotypes without introducing major baseline imbalances, with age showing the clearest difference. The four-gene score was positively associated with the ssGSEA composite, and Immunity-H largely overlapped with ISS2 (Fig. S8). These findings clarify how global immune-activity strata (Immunity-H/L) map onto the four-gene endotypes (ISS1/ISS2). Within ISS2, the hydrocortisone group had higher baseline severity than the no-hydrocortisone group—higher body temperature (37.8 vs 37.4 °C, P = 0.0333), higher APACHE II (24 vs 18, P = 0.0168), and more septic shock (95.2% vs 69.2%, P = 0.0305)—and exhibited higher 28-day and 90-day mortality (52.4% vs 11.5%, P = 0.0036; 61.9% vs 11.5%, P = 0.0005; Table 2). For thymosin in ISS2, several inflammatory/severity markers and ICU length of stay were higher in the treated group, with higher 90-day mortality (57.1% vs 24.2%, P = 0.045; 28-day not significant; Table 3). Given these baseline imbalances, associations between hydrocortisone use and outcomes within ISS2 should be interpreted cautiously and not as causal. Logistic regression analysis revealed that treatment with hydrocortisone (OR = 12.46 [3.11, 65.72], P = 0.0009) or thymosin (OR = 4.17 [1.13, 16.51], P = 0.0347) in ISS2 was associated with higher 90-day mortality (Fig. 3B and C). As a robustness check, Bayesian logistic regression with weakly informative priors yielded directionally consistent effects; full posterior summaries are provided in the Supplement (Table S7, hydrocortisone; Table S8, thymosin). Whereas hydrocortisone was also associated with higher 28-day mortality in ISS2 (OR = 8.43 [2.12, 43.72], P = 0.0047; Table S1). In models including a treatment × endotype term, hydrocortisone showed ISS2-specific risk elevation (HR_ISS2 = 7.44) versus ISS1 (HR_ISS1 = 2.02), with a directionally positive but underpowered interaction (Wald P = 0.109); thymosin showed weaker and non-significant interaction (Wald P = 0.454), with HR_ISS2 = 2.52 and HR_ISS1 = 1.37 (details in Tables S9–S12).

**Table 2:** ISS2 subgroup: baseline characteristics and outcomes by hydrocortisone treatment (Hydrocortisone vs No hydrocortisone)

	Hydrocortisone (n = 21)	No Hydrocortisone (n = 26)	P value
Demographics			
Gender (females)^*^	8 (38.10)	13 (50.00)	0.4144^a^
Age (years)^#^	73 (69, 81)	78 (66, 85)	0.5346^b^
BMI^#^	24.2 (20.8, 26.1)	23.4 (22.0, 24.7)	0.5069^b^
Vital Signs			
Body temperature (°C)^#^	37.8 (37.4, 38.4)	37.4 (36.9, 37.7)	0.0333^b^
Severity Scores			
SOFA score^#^	11 (9, 14)	10 (9, 12)	0.2309^b^
APACHE II^#^	24 (18, 29)	18 (15, 21)	0.0168^b^
Clinical Laboratory Results			
Blood pH^#^	7.38 (7.31, 7.42)	7.38 (7.31, 7.40)	0.6668^b^
PaO2/FiO2^#^	265.3 (169.6, 305.7)	245.9 (171.4, 293.4)	0.4384^b^
Lymphocyte (G/L)^#^	0.58 (0.46, 0.72)	0.61 (0.36, 0.82)	0.9704^b^
Neutrophil (G/L)^#^	13.07 (9.10, 16.07)	11.03 (5.76, 15.79)	0.3126^b^
Monocyte (G/L)^#^	0.46 (0.20, 0.62)	0.52 (0.13, 0.72)	0.9620^b^
Respiratory support			
MV^*^	19 (90.48)	21 (80.77)	0.4364^c^
Complications / Prognosis			
Septic shock^*^	20 (95.24)	18 (69.23)	0.0305^c^
Pulmonary infection^*^	7 (33.33)	5 (19.23)	0.3258^c^
LOS in ICU (days)^#^	5 (3, 12)	4 (3, 9)	0.3278^b^
28-day mortality^*^	11 (52.38)	3 (11.54)	0.0036^c^
90-day mortality^*^	13 (61.90)	3 (11.54)	0.0005^c^

^*^Frequency (%)

^#^Median (IQR)

*a* Chi-square test

*b* Mann–Whitney U test

*c* Fisher's exact Test

*MV* mechanical ventilation, *LOS* length of stay, *SOFA* Sequential organ failure assessment

**Table 3:** ISS2 subgroup: baseline characteristics and outcomes by thymosin treatment (Thymosin vs No thymosin)

	Thymosin (n = 14)	No Thymosin (n = 33)	P value
Demographics			
Gender (females)^*^	3 (21.43)	18 (54.55)	0.0551^c^
Age (years)^#^	78 (72, 84)	74 (65, 85)	0.3352^b^
BMI^#^	21.4 (19.7, 25.0)	23.6 (22.7, 25.4)	0.1488^b^
Vital Signs			
Body temperature (°C)^#^	37.9 (37.4, 38.7)	37.5 (36.8, 37.8)	0.0417^b^
Severity Scores			
SOFA score^#^	12 (9, 12)	11 (9, 12)	0.4623^b^
APACHE II^#^	24 (18, 29)	18 (16, 22)	0.1061^b^
Clinical Laboratory Results			
Blood pH^#^	7.37 (7.30, 7.41)	7.38 (7.31, 7.40)	0.7168^b^
PaO2/FiO2^#^	225.4 (167.7, 299.0)	256.7 (197.3, 305.7)	0.6538^b^
Lymphocyte (G/L)^#^	0.61 (0.52, 0.85)	0.53 (0.29, 0.77)	0.1775^b^
Neutrophil (G/L)^#^	13.33 (11.78, 20.60)	10.78 (5.56, 15.09)	0.0385^b^
Monocyte (G/L)^#^	0.61 (0.47, 1.00)	0.25 (0.12, 0.58)	0.0150^b^
Respiratory support			
MV^*^	13 (92.86)	25 (75.76)	0.2445^c^
Complications / Prognosis			
Septic shock^*^	11 (78.57)	27 (81.82)	> 0.9999^c^
Pulmonary infection^*^	5 (35.71)	7 (19.23)	0.4653^c^
LOS in ICU (days)^#^	8 (4, 12)	4 (2, 7)	0.0425^b^
28-day mortality^*^	6 (42.86)	8 (24.24)	0.2966^c^
90-day mortality^*^	8 (57.14)	8 (24.24)	0.0447^c^

^*^ Frequency (%)

^#^ Median (IQR)

*a* Fisher's exact Test

*b* Mann–Whitney U test;

*MV* mechanical ventilation, *LOS* length of stay, *SOFA* Sequential organ failure assessment

Furthermore, propensity score matching (PSM) was conducted to balance baseline characteristics among patients in different subgroups. This approach aimed to minimize confounding biases and enhance comparability between subgroups, enabling a more robust assessment of the effects within each subgroup. A total of 72 matched samples were included in the logistic regression analysis. The model for ISS1 did not converge during the fitting process, likely because of the limited sample size. In contrast, the ISS2 model converged successfully, showing that hydrocortisone use was associated with higher mortality at 90 days (P = 0.0268) and 28 days (P = 0.0373) in ISS2. These findings support our key conclusions regarding the impact of hydrocortisone. Despite the poor model fit for ISS1, the reliability and scientific validity of the conclusions drawn from ISS2 remain unaffected. Within ISS2, within-stratum PSM comparing hydrocortisone vs. no treatment yielded 6 matched pairs (N = 12). In the matched sample, Firth-penalized logistic regression demonstrated: 90-day mortality: OR = 47.7 [2.94–7830.01], P = 0.0041; 28-day mortality: OR = 23.4 [1.59–3579.50], P = 0.0188 (Table S4). The direction of effect was consistent with the primary analysis. Given the limited sample size and potential separation, the wide confidence intervals warrant cautious interpretation. These findings suggest that accurate stratification of sepsis could improve the effectiveness of immunotherapy in clinical trials.

## Discussion

This study developed and identified a gene panel of immune status in sepsis. Stratification of sepsis based on the ISS-gene panel revealed that hydrocortisone or thymosin therapy was associated with higher mortality within ISS2 subgroup. These findings suggest that direct stratification of sepsis according to immune status contributes to the precise application of immunotherapy. Our findings are consistent with those of previous studies, which reported that patients with SRS2/IA-P endotypes had a significantly higher mortality rate when receiving corticosteroids [16, 19]. However, the patient stratification for steroid treatment response in these studies was based on RNA-seq data, whereas we used RT-PCR, which is more suitable for clinical practice. This approach could reduce the time required for immune stratification of patients to just a few hours. In addition, the development of sepsis biomarkers aims to achieve accurate stratification with fewer markers, and the use of dozens of genes as stratification markers could pose a challenge for future potential rapid diagnostic developments. We propose a streamlined four-gene tool for assessing immune status in sepsis. Our results further expand the application of immune status-based stratification in immunotherapy, and the differential response to thymosin could be extended to other immunotherapies. Notably, the roles of hydrocortisone and thymosin in sepsis immunotherapy are not entirely consistent, and the mechanistic differences between the different drugs in ISS stratification remain to be further explored.

The primary limitation of this study is that the clinical immunotherapy validation was conducted in a single-center, double-blind, randomized cohort. Another limitation is that immune-cell fractions were estimated via the CIBERSORT deconvolution algorithm, which may not fully capture peripheral-blood immune infiltration. Taken together, our data support the concept that a streamlined four-gene panel can reflect sepsis immune status and may help identify subgroups with differential responses to hydrocortisone or thymosin, and future work could develop rapid bedside assays to support ICU decision-making. However, because the randomized, double-blind cohort (n = 99) was underpowered for formal treatment × endotype testing, our interaction analyses were exploratory and imprecise (hydrocortisone: Wald P = 0.109; thymosin: Wald P = 0.454), although stratum-specific hazard ratios suggested a stronger hydrocortisone effect within ISS2. Together with the single-center setting and the use of deconvolution-based cell fractions, these constraints limit generalizability beyond similar ICU populations. Adequately powered multicenter studies with prespecified interaction hypotheses, endotype-stratified randomization, and sample-size calculations for interaction effects are warranted to confirm clinical utility.

We propose promoting a paradigm shift in sepsis biomarker research from diagnostic and prognostic approaches to addressing specific clinical problems. The immune endotype of a patient is crucial for the precise treatment of sepsis, [20, 21] and identifying the immune status of sepsis should be a research priority in this field.

## Supplementary Information

Additional file 1.

## Data Availability

Public datasets analyzed in this study are available from GEO under accession numbers GSE65682 and GSE154918. The de-identified clinical dataset from the Zhongda Hospital cohort is available from the corresponding author on reasonable request, subject to institutional approvals and a data transfer agreement. Analysis code used for ssGSEA/WGCNA and machine-learning model evaluation is available from the authors on reasonable request.
