# Supplementary material for: Development of a gene panel for immune status assessment in sepsis
Source: Ann Intensive Care. 2025 Oct 27;15:170. doi: 10.1186/s13613-025-01594-1 (PMC12554855; doi:10.1186/s13613-025-01594-1)
Supplement: Supplementary file 1 — Additional file 1. [file 13613_2025_1594_MOESM1_ESM.pdf]

Supplementary Materials: Development of a gene panel for immune status assessment in sepsis

Author

1) Chao Gao[1]
2) Xinxing Lu[1]
3) Ying Tang[1]
4) Yunhui Ni[1]
5) Hanbing Chen[1]
6) Xiaojing Wu[1]
7) Xing Zhou[1]
8) Yi Yang[1]
9) Ling Liu[1]
10) Jie Chao[2*]
11) Jianfeng Xie[1*]
12) Haibo Qiu[1]

Contents

S1 Difference validation between Immunity_H and Immunity_L……………………………………….7

S2 Identification of immune status in sepsis-related module…………………………………………….8

S3 Identification of enriched expression of the ISS gene panel using single-cell RNA sequencing……..9

S4 Validation of the ISS gene panel expression in mouse CLP model…………………………………10

S5 Correlation analysis of the ISS gene panel with the clinical parameters (NK cell percentage and monocyte HLA-DR+ percentage) ……………………………………….……………………...11

S6 Identifying K=2 as the optimal number of clusters from consensus clustering CDF analysis………12

S7 Prediction of multi-organ failure by the ISS gene panel……………………………………………...13

S8 Relationship between global immune activity and four-gene ISS endotypes..………….…………..14

S9 Propensity-score overlap and balance diagnostics for ISS1 vs ISS2 (between-group matching)…...15

S10 Robustness of the discovery-stage dichotomy and continuity of immune status…………………..16

Table S1 Differential treatment response to hydrocortisone or thymidine in ISS subgroups…….……..17

Table S2 External validation: AUC, thresholds, and threshold-based metrics……………………......……18

Table S3 Confusion matrices and exact binomial 95% CIs at the stated thresholds……………....……19

Table S4 Effect estimates in the matched sample……………………………………………......……20

Table S5 Bayesian logistic regression in ISS2: posterior summaries for hydrocortisone……………..……21

Table S6 Bayesian logistic regression in ISS2: posterior summaries for thymosin…………..…....………22

Table S7 Cox model with treatment × endotype interaction (hydrocortisone × ISS); 90-day mortality…23

Table S8 Stratum-specific hazard ratios for hydrocortisone within ISS1 and ISS2 derived from the interaction model; 90-day mortality……………………………………………………………......……24

Table S9 Cox model with treatment × endotype interaction (thymosin × ISS); 90-day mortality………25

Table S10 Stratum-specific hazard ratios for thymosin within ISS1 and ISS2 derived from the interaction model; 90-day mortality………………………………………………………………..…....……26

Figure S1. Difference validation between Immunity_H and Immunity_L

(A) -SNE plots are used to visualize the distribution of different subtypes, clearly distinguishing the clustering patterns (Figure S1A). (B, C)The abundance of various immune cell populations across two subtypes, highlighting significant differences in immune cell infiltration. (D) astly, the expression levels of multiple HLA genes across two of the identified subtypes, showing differential HLA expression between the subgroups.

Figure S2. WGCNA Module and ISS Related Genes Analysis

To identify the most appropriate soft-thresholding power, we performed scale-free topology analysis, selecting the soft threshold where the network topology best fits a scale-free distribution. Subsequently, we applied hierarchical clustering to all genes to identify gene modules based on their co-expression patterns. In addition, to identify genes and pathways associated with the immune status in sepsis, we selected the dark red module for further analysis using Gene Ontology (GO) and Kyoto Encyclopedia of Genes and Genomes (KEGG). The genes within this module were predominantly involved in immune cell-mediated immunity and cytotoxicity .

Figure S3. Identification of Cell Types Expressing the ISS Gene Panel

Analysis of single-cell datasets indicated that NK and T cells influence the immune status of patients with sepsis.

Figure S4. Analysis of ISS Gene Panel Expression Changes in the Animal Model

The four selected genes were downregulated in the CLP model and their expression levels correlated with disease severity.

Figure S5. Correlation analysis of the ISS gene panel with the clinical parameters

To explore the relationship between the ISS gene panel and traditional immune markers, we analyzed RNA-seq data from patients with sepsis at our hospital (n = 19).

Figure S6. Determining Optimal Cluster Number: K=2 from CDF Stability Analysis

The CDF plot shows that as K increases, the CDF curve becomes more stable. At K=2, the CDF reaches a plateau with minimal changes in the cumulative distribution, indicating that the consensus matrix is highly stable and well-separated at this point. Beyond K=2, the improvements in the CDF curve are marginal, suggesting that increasing the number of clusters does not significantly improve the clustering stability. Therefore, K=2 is the optimal choice for clustering based on the CDF analysis.

Figure S7. Prediction of Multi-organ Failure by the ISS Gene Panel

We further sought to utilize this gene panel, along with patient age and sex, to predict multi-organ failure three days after ICU admission. Multi-organ failure is defined as a SOFA score of ≥1 for more than two systems on day three.

Figure S8. Relationship between global immune activity (Immunity-H/L) and four-gene ISS endotypes (ISS1/ISS2)

A Scatterplot of the four-gene composite (z-sum of TBX21, GNLY, PRF1, IL2RB) versus the composite immune-activity score (mean of z-scored ssGSEA features). Dashed lines mark median-based

dichotomies used to define Immunity-H/L and ISS2/ISS1. A strong positive monotonic association is observed (Spearman $\rho = 0.73$; $P<2\times10^{-16}$), indicating that higher global immune activity corresponds to higher four-gene expression. B 2×2 cross-tabulation (heat map with counts and row-wise percentages) comparing Immunity-H/L with ISS1/ISS2 shows predominant alignment of Immunity-H with ISS2 and Immunity-L with ISS1, supporting the intended two-tier pipeline (global immune activity → four-gene clinical endotyping).

Figure S9. Propensity-score overlap and balance diagnostics for ISS1 vs ISS2 (between-group matching)

(A) Histograms (or kernel densities) of estimated propensity scores for ISS1 and ISS2 before matching (top) and after 1:1 nearest-neighbor matching (bottom). After matching, the two distributions exhibit substantially greater overlap within the region of common support, indicating improved comparability. The matched sample comprises 36 pairs (N=72); observations outside common support were discarded.

Figure S10. Robustness of the discovery-stage dichotomy and continuity of immune status

(A) Continuous modeling of immune activity: scatter of the four-gene composite (z-sum of TBX21, GNLY, PRF1, IL2RB) versus the composite immune-activity score (mean of z-scored ssGSEA features). Linear fit versus natural cubic spline are compared; Linear $R^2 = 0.535$, AIC(linear) = 3572, AIC(spline) = 3569.8, likelihood-ratio P = 0.043, indicating only limited incremental fit from the spline. (B) Agreement between the median split and unsupervised k-means (k=2): confusion heat map with counts and row-wise percentages; Cohen's $\kappa = 0.908$ and Adjusted Rand Index (ARI) = 0.824, showing excellent concordance. (C) Distribution of the immune-activity score with alternative thresholds. Dashed line = median; dotted lines = k-means centers; dot-dash lines = Q1 & Q3. The density profile shows no obvious natural cutoff, supporting the reasonableness of the median-based dichotomy.

Table S1. Differential treatment response to hydrocortisone or thymidine in ISS subgroups; 28-day mortality

Table S2. External validation: AUC, thresholds, and threshold-based metrics

Abbreviations: AUC, area under the ROC curve; CI, confidence interval; Se, sensitivity; Sp, specificity; Acc, accuracy.

Table S3. Confusion matrices and exact binomial 95% CIs at the stated thresholds

Abbreviations: TN, true negative; FP, false positive; FN, false negative; TP, true positive; PPV, positive predictive value; NPV, negative predictive value; CI, confidence interval.

Table S4. Effect estimates in the matched sample

ORs (95% CIs) and P values from Firth models; with matched-pair counts and matched-sample size.

To reduce confounding within strata and obtain interpretable effect estimates, we performed within-stratum PSM in ISS2, comparing hydrocortisone vs. no treatment. Propensity scores were estimated via multivariable logistic regression with prespecified baseline covariates (age, sex, SOFA, APACHE II, septic shock). The estimand was the average treatment effect on the treated (ATT). We used 1:1 nearest-neighbor matching without replacement or optimal matching on the logit PS with a 0.20×SD(logitPS) caliper and trimmed for common support if needed. The primary outcomes were 90- and 28-day mortality.

Given the small matched sample and the risk of quasi/complete separation, we prespecified Firth-penalized logistic regression as the primary analysis in the matched sample, reporting ORs with 95% CIs.

Table S5. Bayesian logistic regression in ISS2: posterior summaries for hydrocortisone

Posterior summaries from a Bayesian logistic regression (logit link) for 90-day mortality within the ISS2 stratum. Weakly informative priors were used: treatment coefficient ~ Student-t(df=3, 0, 2.5); intercept ~ Student-t(df=3, 0, 10). Reported metrics include the posterior median OR with 95% credible interval (CrI), Pr(OR>1), g-computed posterior standardized risks (exposed p_1, unexposed p_0; each with 95% CrIs), and the risk difference (RD = $p_1 - p_0$, 95% CrI). Model adequacy was assessed via posterior predictive checks and LOO-CV. Abbreviations: OR, odds ratio; CrI, credible interval; RD, risk difference.

Table S6. Bayesian logistic regression in ISS2: posterior summaries for thymosin

Same modeling framework and priors as Table S5. The table reports posterior median ORs (95% CrIs), Pr(OR>1), posterior standardized risks (p_1, p_0; with 95% CrIs), and the risk difference (RD, 95% CrI) for thymosin within ISS2. Model adequacy was evaluated using posterior predictive checks and LOO-CV. Abbreviations and rounding notes as in Table S5.

Table S7. Cox model with treatment × endotype interaction (hydrocortisone × ISS); 90-day mortality

Cox proportional hazards model including treatment (hydrocortisone vs no hydrocortisone), endotype (ISS2 vs ISS1), and their interaction term (treatment×ISS). Entries are hazard ratios (HR) with 95% confidence intervals and two-sided Wald P-values. The interaction row (hyd:ISS) tests whether the hydrocortisone effect differs between ISS2 and ISS1. Likelihood-ratio tests (models with vs without the interaction) are reported in the text; proportional-hazards assumptions were checked using Schoenfeld residuals. Endpoint: time to 90-day all-cause mortality; ties handled by Efron method. Abbreviations: HR, hazard ratio; CI, confidence interval.

Table S8. Stratum-specific hazard ratios for hydrocortisone within ISS1 and ISS2 derived from the interaction model; 90-day mortality

Stratum-specific Hyd vs NoHyd effects estimated from the interaction model: HR_ISS1 = exp(β_treat) and HR_ISS2 = exp(β_treat + β_treat×ISS); 95% CIs computed via the delta method; two-sided Wald P-values shown. These estimates correspond to the within-endotype hydrocortisone association and complement the formal interaction test in the coefficient table. Abbreviations: HR, hazard ratio; CI, confidence interval.

Table S9. Cox model with treatment × endotype interaction (thymosin × ISS); 90-day mortality

Cox proportional hazards model including treatment (thymosin vs no thymosin), endotype (ISS2 vs ISS1), and their interaction term (treatment×ISS). Entries are hazard ratios (HR) with 95% confidence intervals and two-sided Wald P-values. The interaction row (treat:ISS) tests whether the thymosin effect differs between ISS2 and ISS1. A likelihood-ratio test comparing models with vs without the interaction is reported in the text; the proportional-hazards assumption was assessed using Schoenfeld residuals. Endpoint: time to 90-day all-cause mortality; ties handled by Efron method. Abbreviations: HR, hazard ratio; CI, confidence interval; ISS, immune status subtype.

Table S10. Stratum-specific hazard ratios for thymosin within ISS1 and ISS2 derived from the interaction model; 90-day mortality

Stratum-specific thymosin vs no thymosin effects derived from the interaction model: $HR\_ISS1 = \exp(\beta\_treat)$ and $HR\_ISS2 = \exp(\beta\_treat + \beta\_treat \times ISS)$. 95% CIs were obtained via the delta method; two-sided Wald P-values are shown. These estimates complement the formal interaction test by quantifying the treatment association within each endotype. Given limited events, results should be interpreted as exploratory. Abbreviations: HR, hazard ratio; CI, confidence interval; ISS, immune status subtype.

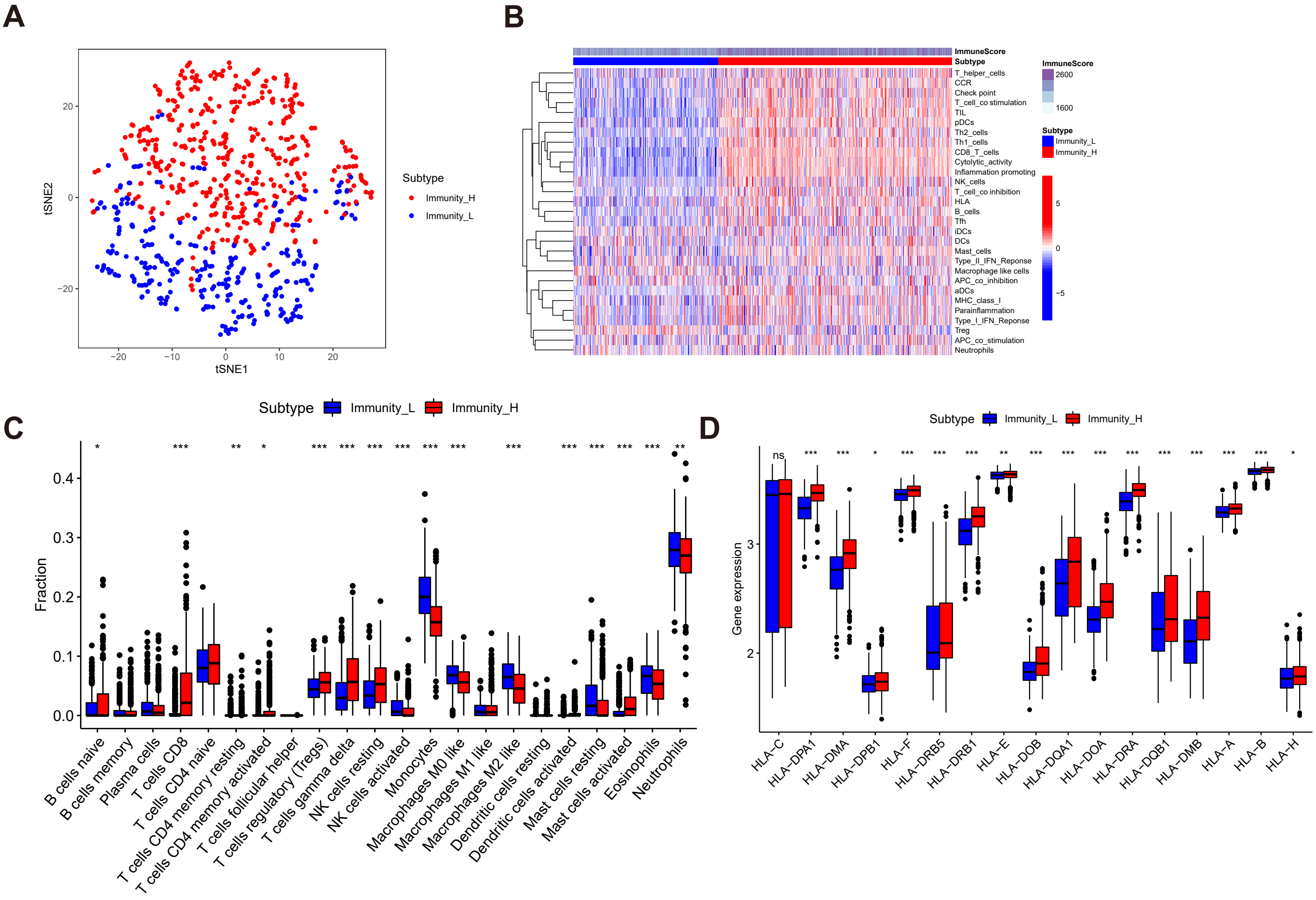

Suppl. fig 1 Difference validation between Immunity_H and Immunity_L. A Displaying subtypes with t-SNE plots. B and C Immune cell abundances in different subtypes. D Multiple HLA expression levels in two subtypes. *P < 0·05, **P < 0·01, ***P < 0·001

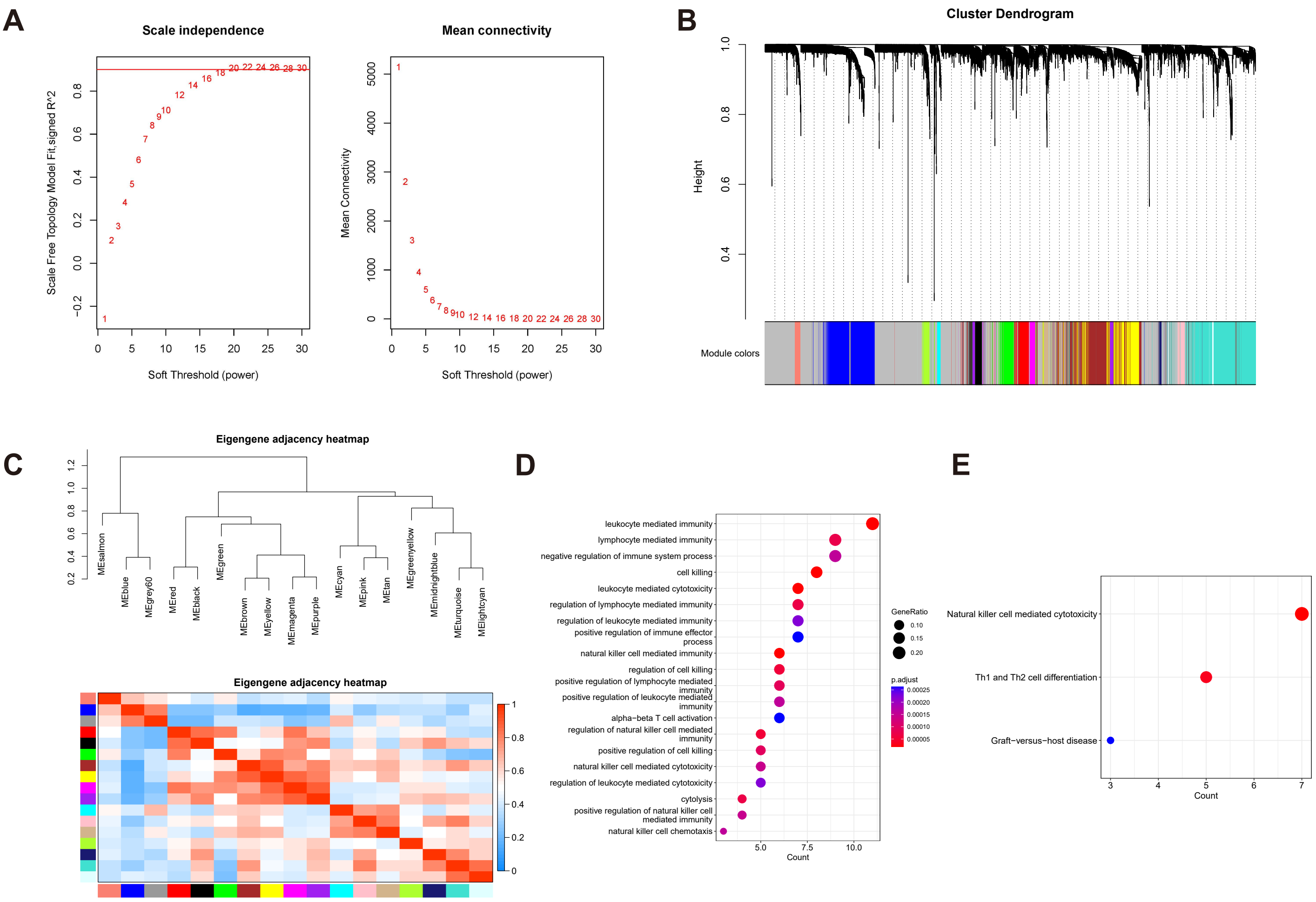

Suppl. fig. 2 Identification of immune status in sepsis-related module. A Analysis of the scale-free fit index for various soft-thresholding powers to determine the feasible soft-thresholding (β = 20, scale-free R2 = 0·92). B The gene cluster dendrogram according to hierarchical clustering analysis of adjacency value difference. C The correlation between different modules. D GO analysis to annotate the gene functions of the dark red module. E Prediction of pathways associated with sepsis immune status based on KEGG Pathway Enrichment Scores.

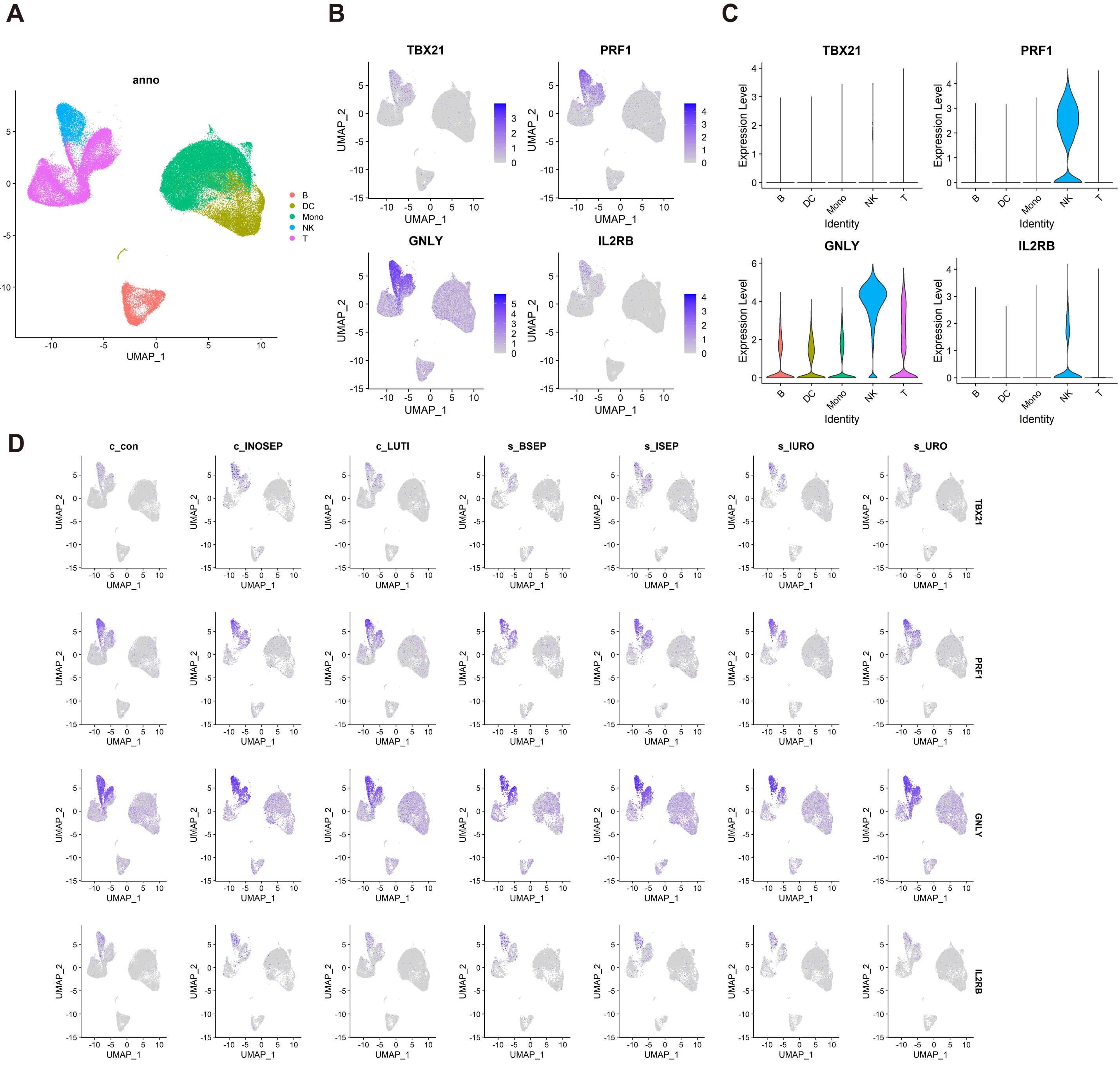

Suppl. fig. 3 Identification of enriched expression of the ISS gene panel using single-cell RNA sequencing. A Definition of immune cell clusters based on clustering their gene expression profiles. B Distribution of the ISS gene panel-positive cells in each cluster. C Enrichment of the ISS gene panel-positive cells in different immune cells. D, E Expression and distribution of the ISS gene panel in each sample type.

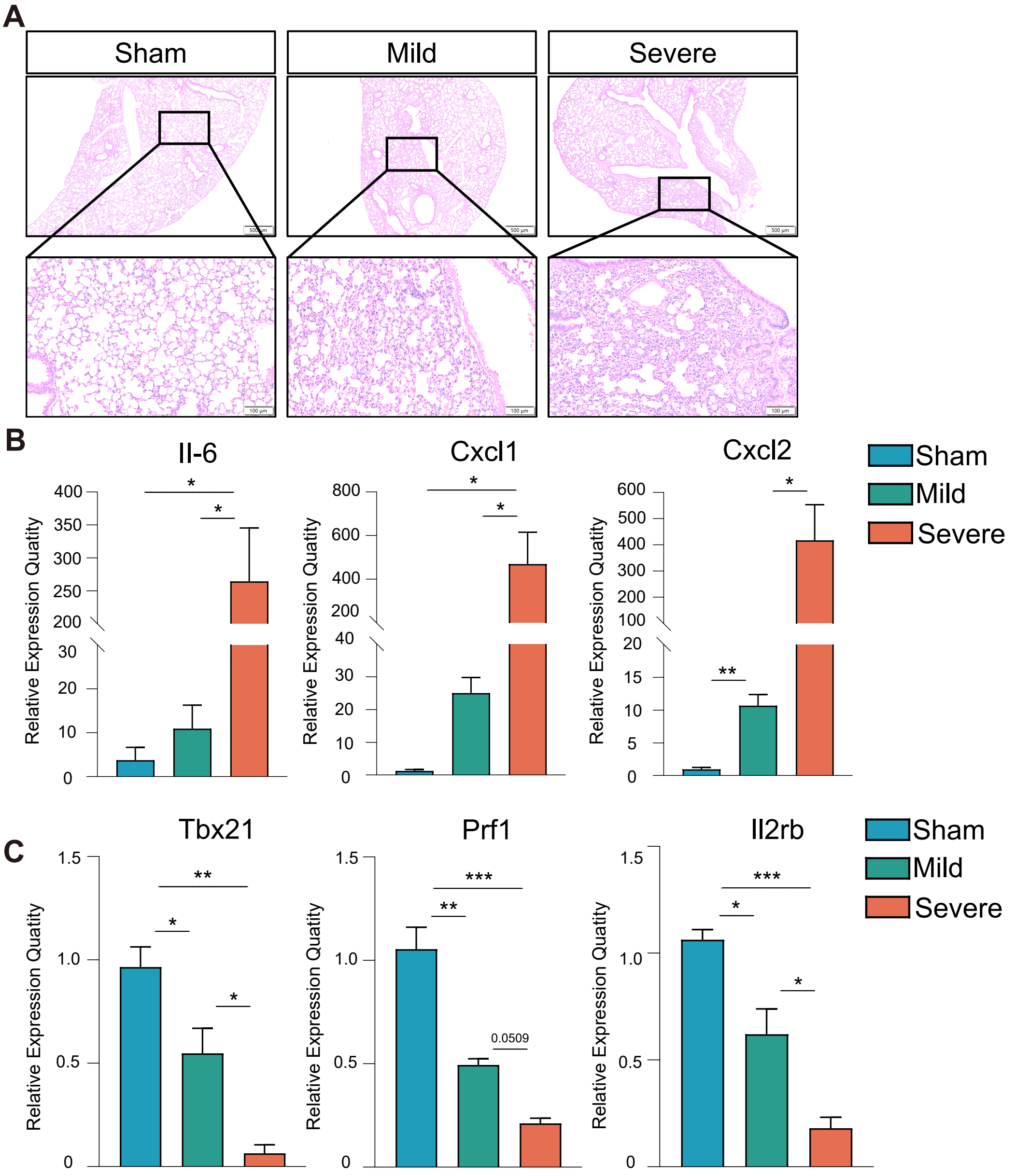

Suppl. fig. 4 Validation of the ISS gene panel expression in mouse CLP model. A, B Different severity levels CLP model was verified through hematoxylin-eosin (H&E) stain and the expression of cytokines/chemokines. C mRNA expression of the three genes in CLP models. (Mice do not express homologs of the human GNLY gene) *P < 0·05, **P < 0·01, ***P < 0·001

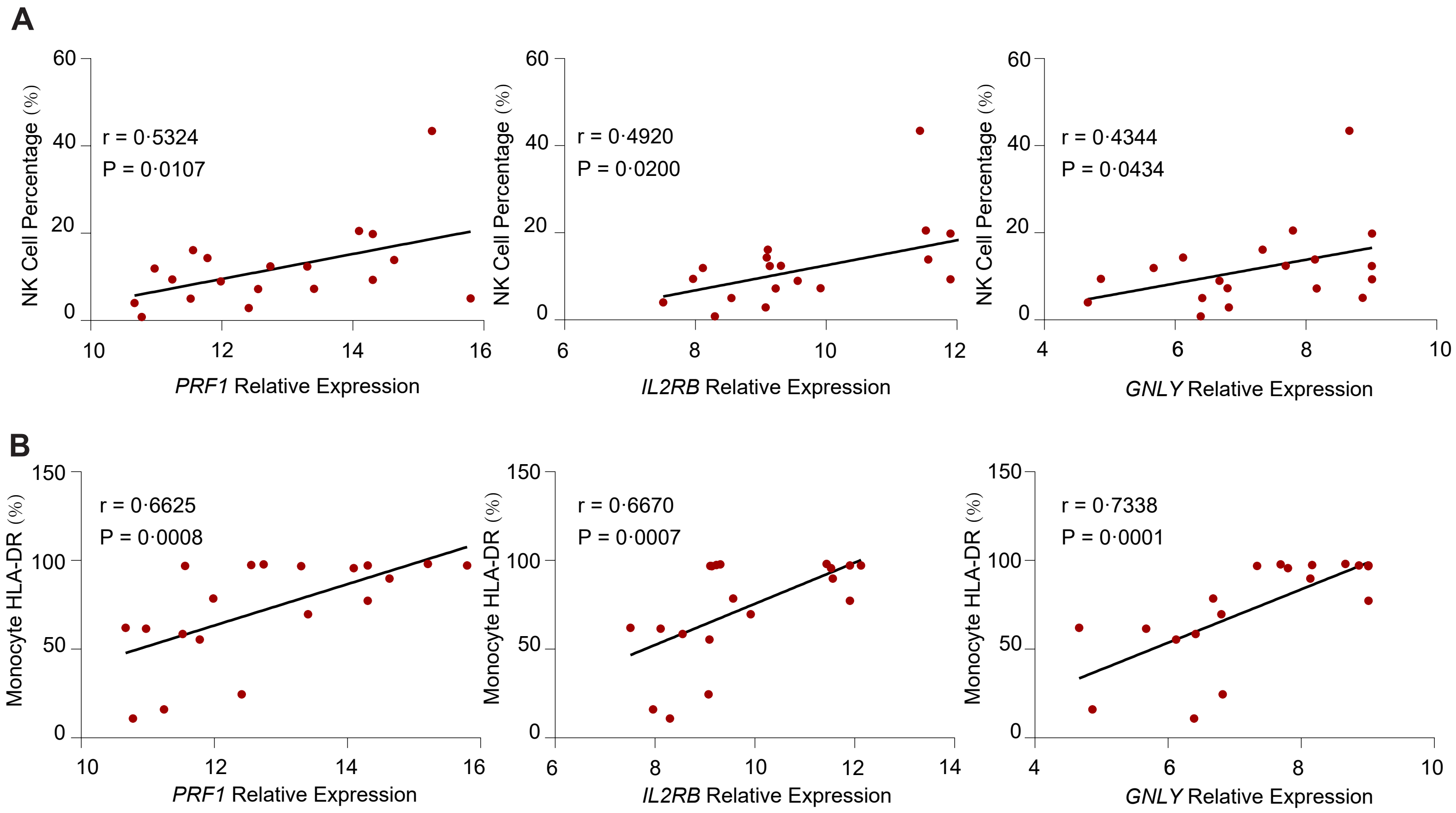

Suppl. fig. 5 Correlation analysis of the ISS gene panel with the clinical parameters (NK cell percentage and monocyte HLA-DR levels). A Correlation between the expression of the three genes (*PRF1*, *IL2RB*, and *GNLY*) and NK cell percentage. B The correlation between the expression of the three genes (*PRF1*, *IL2RB*, and *GNLY*) and monocyte HLA-DR expression. (Data were obtained from our transcriptome database, which unfortunately did not detect TBX21 expression)

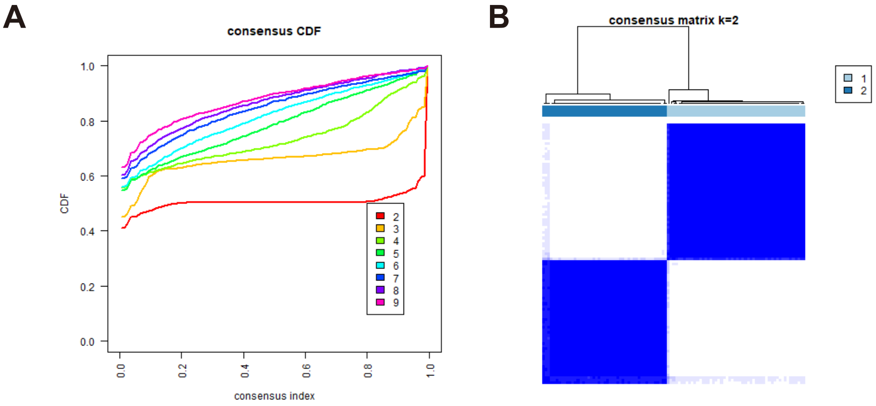

Suppl. fig. 6 Identifying K=2 as the optimal number of clusters from consensus clustering CDF analysis. A, B Based on the CDF analysis, K=2 is identified as the optimal number of clusters for achieving stable and well-separated clustering.

A

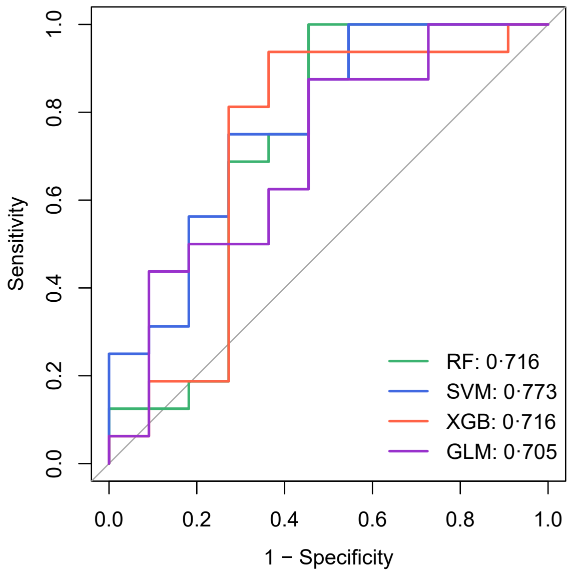

Suppl. fig. 7 Prediction of multi-organ failure by the ISS gene panel. A Four machine-learning algorithms to predict >2 organ failures.

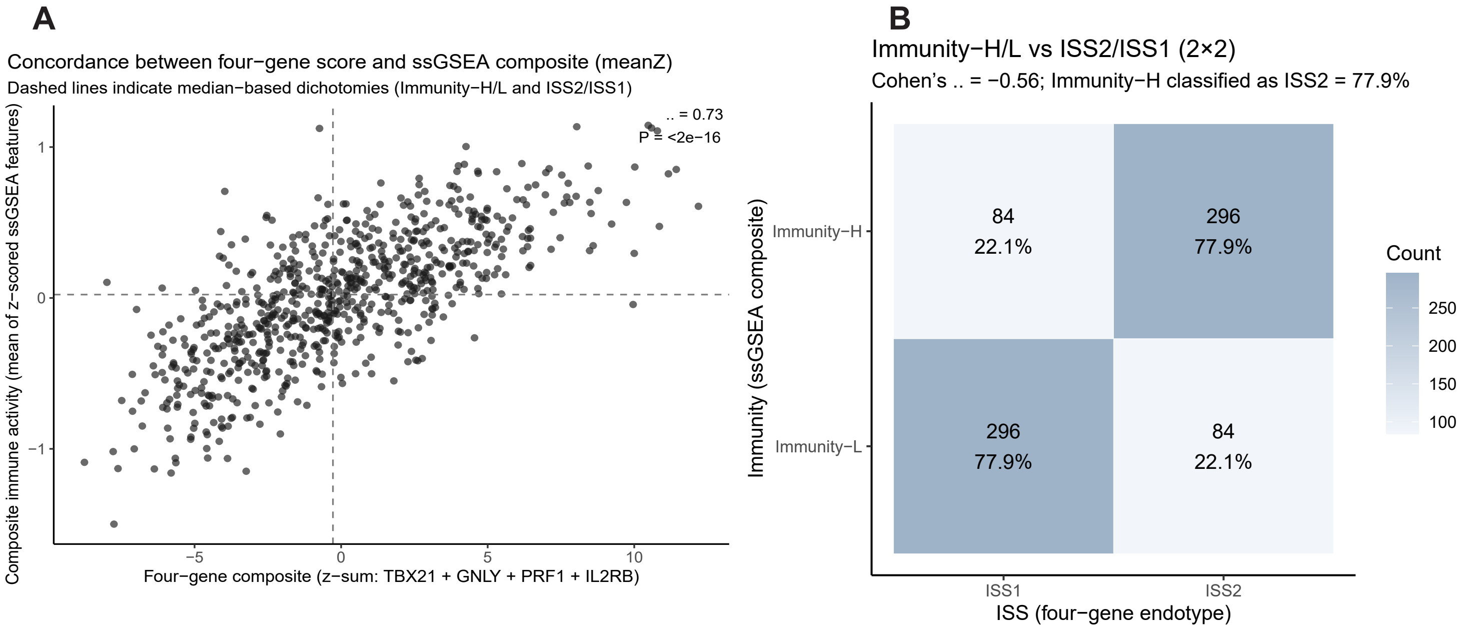

Suppl. fig. 8 Bridging global immune activity and four-gene clinical endotypes. A Scatterplot of the four-gene composite (z-sum of TBX21, GNLY, PRF1, IL2RB) versus the composite immune-activity score (mean of z-scored ssGSEA features). Dashed lines mark median-based dichotomies used to define Immunity-H/L and ISS2/ISS1. A strong positive monotonic association is observed (Spearman $\rho = 0.73$; $P<2\times10^{-16}$), indicating that higher global immune activity corresponds to higher four-gene expression. B 2×2 cross-tabulation (heat map with counts and row-wise percentages) comparing Immunity-H/L with ISS1/ISS2 shows predominant alignment of Immunity-H with ISS2 and Immunity-L with ISS1, supporting the intended two-tier pipeline (global immune activity - four-gene clinical endotyping). Abbreviations: ssGSEA, single-sample Gene Set Enrichment Analysis; ISS, immune status subtype.

A

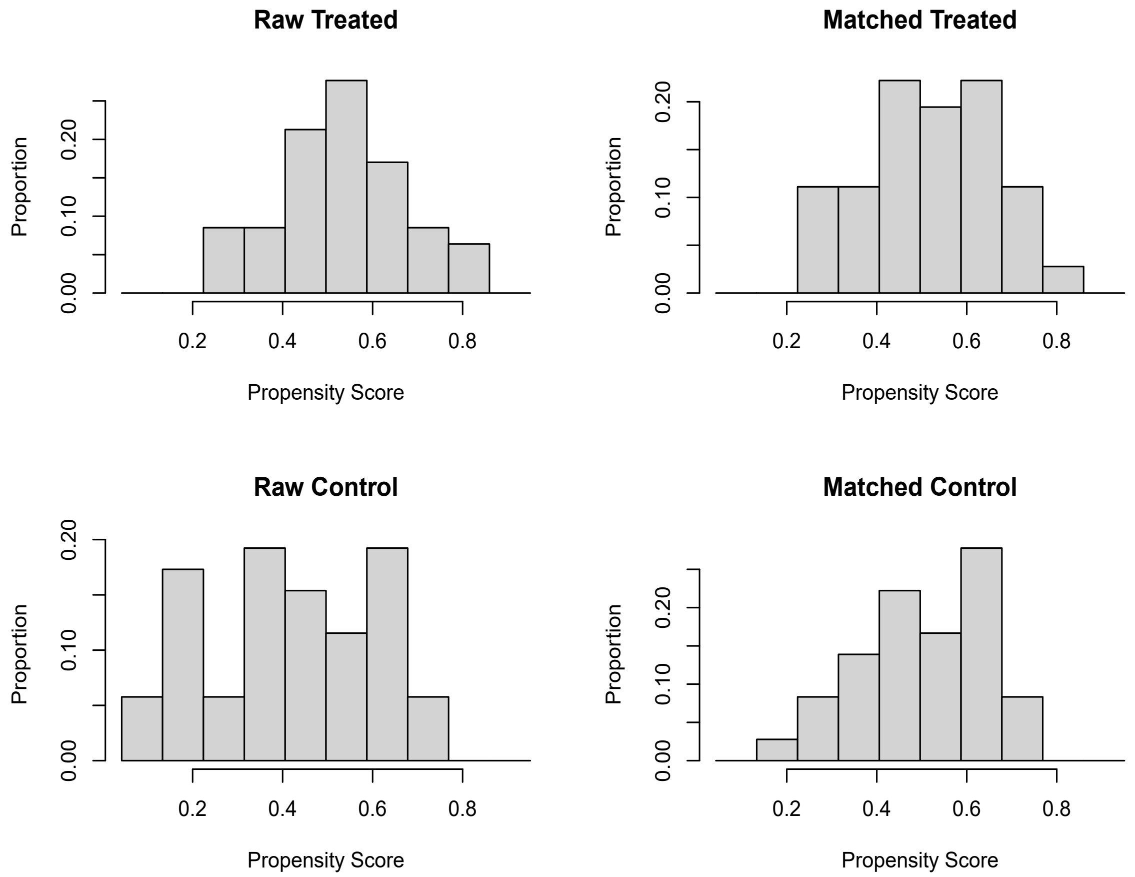

Suppl. fig. 9 Propensity-score overlap and balance diagnostics for ISS1 vs ISS2 (between-group matching). A Histograms (or kernel densities) of estimated propensity scores for ISS1 and ISS2 before matching (top) and after 1:1 nearest-neighbor matching (bottom). After matching, the two distributions exhibit substantially greater overlap within the region of common support, indicating improved comparability. The matched sample comprises 36 pairs (N=72); observations outside common support were discarded.

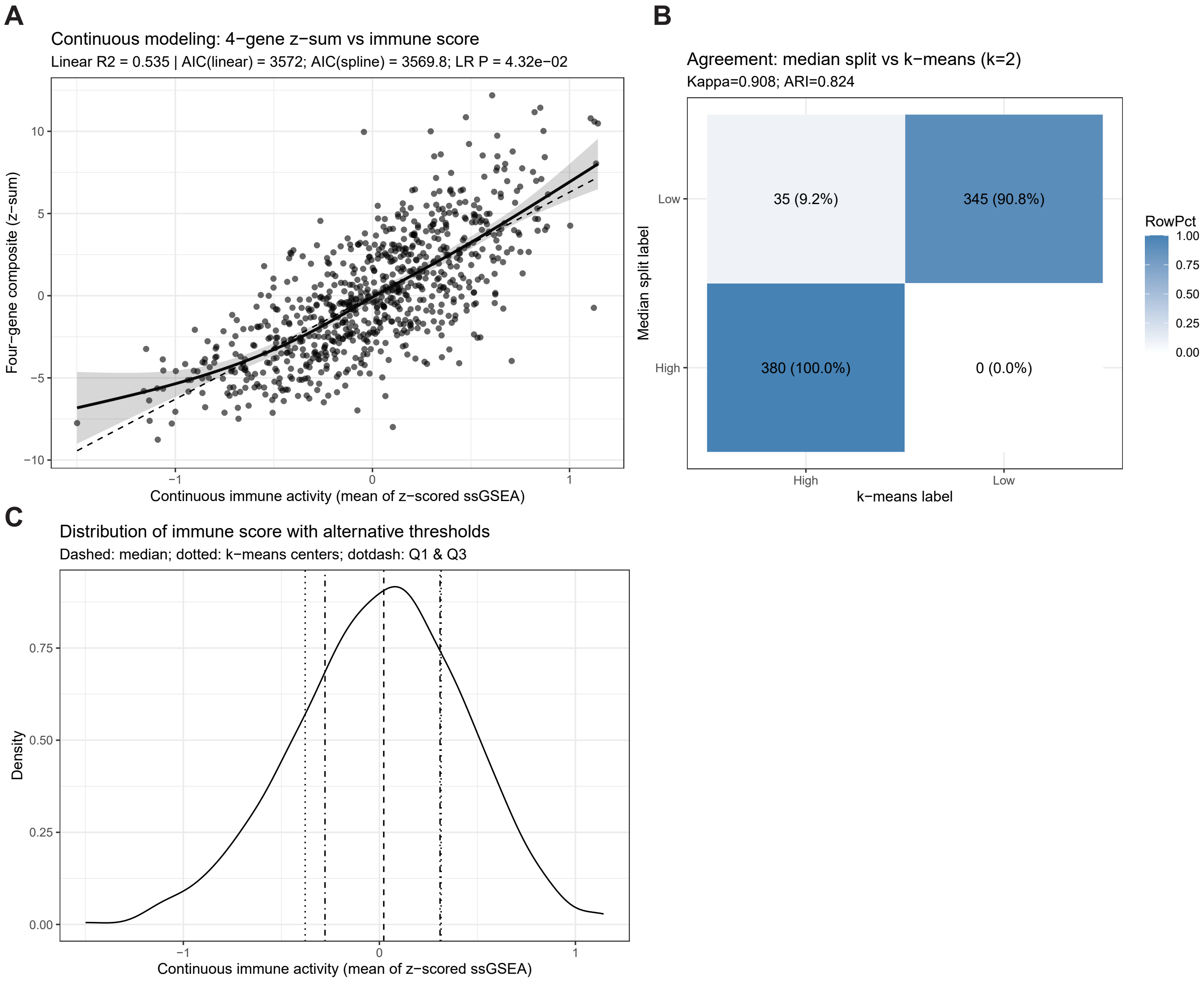

Suppl. fig. 10 Robustness of the discovery-stage dichotomy and continuity of immune status. A Continuous modeling of immune activity: scatter of the four-gene composite (z-sum of TBX21, GNLY, PRF1, IL2RB) versus the composite immune-activity score (mean of z-scored ssGSEA features). Linear fit versus natural cubic spline are compared; Linear R^2 = 0.535, AIC(linear)=3572, AIC(spline)=3569.8, likelihood-ratio P = 0.043, indicating only limited incremental fit from the spline. B Agreement between the median split and unsupervised k-means (k=2): confusion heat map with counts and row-wise percentages; Cohen's κ = 0.908 and Adjusted Rand Index (ARI) = 0.824, showing excellent concordance. C Distribution of the immune-activity score with alternative thresholds. Dashed line = median; dotted lines = k-means centers; dot-dash lines = Q1 & Q3. The density profile shows no obvious natural cutoff, supporting the reasonableness of the median-based dichotomy.

Table S1. Differential treatment response to hydrocortisone or thymidine in ISS subgroups; 28-day mortality

	Odds ratio	95% confidence intervals	P Value
Treatment with hydrocortisone in ISS1	2.89	0.83 to 10.64	0.0990
Treatment with hydrocortisone in ISS2	8.43	2.12 to 43.72	0.0047
Treatment with thymosin in ISS1	3.40	0.69 to 16.92	0.1229
Treatment with thymosin in ISS2	2.34	0.61 to 8.98	0.2075

Table S2 External validation: AUC, thresholds, and threshold-based metrics

Model	AUC (95% CI)	Threshold	Se	Sp	Acc	Se@0.5	Sp@0.5	Acc@0.5
RF	0.891 (0.764–0.991)	0.98	0.432	1	0.49	0.886	0.4	0.837
SVM	0.905 (0.812–0.997)	0.845	0.818	1	0.837	0.909	0.4	0.857
XGB	0.900 (0.810–0.990)	0.974	0.455	1	0.51	0.818	0.8	0.816
GLM	0.909 (0.809–1.000)	0.914	0.523	1	0.571	0.864	0.6	0.837

Abbreviations: AUC, area under the ROC curve; CI, confidence interval; Se, sensitivity; Sp, specificity; Acc, accuracy; RF, random forest; SVM, support vector machine; XGB, extreme gradient boosting; GLM, generalized linear model (logistic regression); Se@0.5/Sp@0.5/Acc@0.5, metrics computed at probability cutoff 0.5; Threshold, probability cutoff applied to the external cohort.

Table S3 Confusion matrices and exact binomial 95% CIs at the stated thresholds

Model	Threshold	TN	FP	FN	TP	Sensitivity(95% CI)	Specificity(95% CI)	PPV	NPV
RF	0.98	5	0	25	19	0.432 (0.283–0.590)	1.000 (0.478–1.000)	1.000 (0.824–1.000)	0.167 (0.056–0.347)
SVM	0.845	5	0	8	36	0.818 (0.673–0.918)	1.000 (0.478–1.000)	1.000 (0.903–1.000)	0.385 (0.139–0.684)
XGB	0.974	5	0	24	20	0.455 (0.304–0.612)	1.000 (0.478–1.000)	1.000 (0.832–1.000)	0.172 (0.058–0.358)
GLM	0.914	5	0	21	23	0.523 (0.367–0.675)	1.000 (0.478–1.000)	1.000 (0.852–1.000)	0.192 (0.066–0.394)

Abbreviations: TN, true negative; FP, false positive; FN, false negative; TP, true positive; PPV, positive predictive value; NPV, negative predictive value; CI, confidence interval; RF, random forest; SVM, support vector machine; XGB, extreme gradient boosting; GLM, generalized linear model (logistic regression); Sensitivity/Specificity (95% CI), exact binomial 95% confidence intervals.

Table S4 Effect estimates in the matched sample

Treatment	Outcome	Model	OR	95% CI (low–high)	P value	Pairs	N (matched)
Hydrocortisone	90-day death	Firth (matched sample)	47.70	2.94 – 7830.01	0.0041	6	12
Hydrocortisone	28-day death	Firth (matched sample)	23.40	1.59 – 3579.50	0.0188	6	12

Table S5. Bayesian logistic regression in ISS2: posterior summaries for hydrocortisone

beta_median	beta_CrI_low	beta_CrI_high	OR_median	OR_CrI_low	OR_CrI_high	Pr_OR_gt1	p1_median	p1_CrI_low	p1_CrI_high	p0_median	p0_CrI_low	p0_CrI_high	RD_median	RD_CrI_low	RD_CrI_high
2.4306	1.1049	4.0894	11.37	3.02	59.71	1	0.606	0.394	0.796	0.121	0.033	0.277	0.476	0.228	0.697

Table S6. Bayesian logistic regression in ISS2: posterior summaries for thymosin

beta_median	beta_CrI_low	beta_CrI_high	OR_median	OR_CrI_low	OR_CrI_high	Pr_OR_gt1	p1_median	p1_CrI_low	p1_CrI_high	p0_median	p0_CrI_low	p0_CrI_high	RD_median	RD_CrI_low	RD_CrI_high
1.3477	0.0638	2.66	3.85	1.07	14.3	0.98	0.553	0.303	0.784	0.245	0.123	0.407	0.303	0.013	0.568

Table S7. Cox model with treatment × endotype interaction (hydrocortisone × ISS); 90-day mortality

term	estimate	std.error	statistic	p.value	conf.low	conf.high
hyd	2.017401	0.50061	1.40191	0.160942	0.756262	5.381612
ISS	0.458495	0.677059	-1.15176	0.249422	0.121625	1.728415
hyd:ISS	3.686263	0.812994	1.604702	0.10856	0.749142	18.13879

Table S8. Stratum-specific hazard ratios for hydrocortisone within ISS1 and ISS2 derived from the interaction model; 90-day mortality

Stratum	HR	CI_low	CI_high	Wald_P
ISS1	2.017401	0.756248	5.381709	0.160942
ISS2	7.436671	2.114258	26.15768	0.001767

Table S9. Cox model with treatment × endotype interaction (thymosin × ISS); 90-day mortality

term	estimate	std.error	statistic	p.value	conf.low	conf.high
thymosin	1.373900193	0.640984571	0.495571291	0.620196912	0.391154318	4.825721346
ISS	0.849489478	0.449408453	-0.36296541	0.716630722	0.352063365	2.049722991
treat:ISS	1.837093791	0.813137119	0.747948713	0.454491093	0.373239637	9.042216476

Table S10. Stratum-specific hazard ratios for thymosin within ISS1 and ISS2 derived from the interaction model; 90-day mortality

Stratum	HR	CI_low	CI_high	Wald_P
ISS1	1.373900193	0.391145288	4.82583275	0.620196912
ISS2	2.523983514	0.945961436	6.734410659	0.064450502
